# An Improved Adaptive Monte Carlo Localization Algorithm Integrated with a Virtual Motion Model

**DOI:** 10.3390/s25082471

**Published:** 2025-04-14

**Authors:** Cili Zuo, Demin Xie, Lianghong Wu, Xiaolong Tang, Hongqiang Zhang

**Affiliations:** 1 School of Information and Electrical Engineering, Hunan University of Science and Technology, Xiangtan 411201, China; zuocl@hnust.edu.cn (C.Z.); 19967632798@163.com (X.T.); hongqiangzhang@hnust.edu.cn (H.Z.); 2 School of Electrical and Engineering Hunan Industry Polytechnic, Changsha 410208, China

**Keywords:** mobile robot localization, adaptive Monte Carlo localization, normal distributions transform, extended Kalman filter

## Abstract

Regarding the issue of high dependency on odometry in the adaptive Monte Carlo localization (AMCL) algorithm, an improved AMCL algorithm based on the normal distributions transform (NDT) and extended Kalman filter (EKF) is proposed. A virtual motion model is introduced into the AMCL framework to enable pose updates even when the robot has not moved. NDT is used for point cloud matching to estimate virtual displacement and calculate virtual control quantities, which are then fed into the motion model to predict and update particle states when the robot has not moved. Additionally, to avoid the negative impacts of encoder errors and wheel slippage on motion state estimation, the EKF algorithm integrates information from the wheel odometer and inertial measurement unit to estimate the robot’s displacement, thereby improving localization accuracy and stability. The performance of the proposed algorithm was experimentally validated in both simulated and real environments and compared with other localization algorithms. Experimental results show that the proposed algorithm can effectively improve localization speed during the cold start phase and enhances localization accuracy and stability throughout the localization process. The proposed method is a potential method for improving the performance of mobile robot localization.

## 1. Introduction

With the rapid development of society, robots are playing an increasingly significant role in human production systems and daily life [1,2]. Among them, mobile robots, as one of the most widely used types of intelligent robots, are extensively employed in logistics, industrial applications, and other fields [3,4,5]. The process by which a mobile robot determines its pose within an environment based on sensor input is referred to as the localization problem [6]. Localization accuracy is a critical factor for achieving precise robot control [7]. For outdoor autonomous mobile robots, the Global Navigation Satellite System (GNSS) is typically used as the primary localization method, supplemented by local sensor systems. However, when satellite signals are obstructed by objects, localization errors can exceed several meters [8]. For indoor mobile robots, satellite signals are severely blocked, necessitating alternative localization methods. To address this, researchers have developed technologies such as infrared [9], Bluetooth [10], Wi-Fi [11], Ultra-Wide Band (UWB) [12], and pseudo-satellites [13]. Nevertheless, these indoor localization methods all have limitations. For example, infrared localization achieves high precision but suffers from weak penetration capabilities and short transmission distances [14]. Bluetooth has a limited range and relatively high costs [15], while Wi-Fi is constrained by coverage limitations, energy consumption, and susceptibility to interference [16]. Furthermore, these methods require the deployment of base stations or devices in the environment, and their signals are easily obstructed, degrading localization performance. In comparison, robot autonomous positioning is less constrained by infrastructure and has lower deployment costs, which has garnered extensive research [17].

Mobile robots can estimate their position and posture using motion state sensors carried by the robots. These sensors include motor encoders, Inertial Measurement Units (IMUs), etc., which capture the wheel speed, as well as the robot’s acceleration, angular velocity, and other motion state information. By performing kinematic calculations, the robot’s position and attitude are updated [18,19]. Although this method is cost-effective and does not rely on external devices, it has poor robustness, large cumulative errors, and generally only enables relative positioning [20]. Simultaneous Localization and Mapping (SLAM) technology allows robots to perceive their surroundings using laser radars or cameras and use the environmental information to draw maps. By referencing the maps, robots can autonomously localize within the environment, reducing the impact of cumulative errors [21]. Environmental information and map information can be matched using the grid method. By dividing the pose space into grids, sensor data are matched with existing grid maps using histogram filtering to update pose information [22]. However, in practical implementation, the performance of grid localization is often limited by the required grid granularity. Coarse grids, due to their high degree of discretization, may result in information loss, negatively impacting the filtering process and affecting localization accuracy and stability. Conversely, fine grids significantly increase computational load, leading to a considerable reduction in algorithm speed. To overcome the drawbacks of the above localization methods, researchers have proposed probabilistic localization methods based on the Markov model [23]. These methods divide the environment into grids and, by combining the robot’s motion state model and observation model, use simple motion and observation models to reduce computational complexity, achieving robust robot pose estimation.

Monte Carlo localization (MCL) is a typical probability-based localization method that predicts the robot’s movement displacement based on odometry data and updates the robot’s pose estimation using sensed environment information and map data [24]. The MCL algorithm can efficiently solve pose tracking and global localization, but it has an issue with the “robot kidnapping” problem. To address this issue, adaptive Monte Carlo localization (AMCL) introduces random particles into the particle set under the assumption that the robot has a small probability of being “kidnapping”. These random particles introduce stochasticity into the model, allowing the robot to recover from the kidnapped state. Even without “kidnapping”, random particles enhance system robustness [25]. Currently, AMCL is a widely used and researched mobile robot localization method. Zhang L et al. treated robot pose as an unknown variable, used sensor-derived environmental data as observations, computed the conditional probability of poses given observations, and optimized this probability to achieve localization [26]. Hatem I et al. proposed generalized Monte Carlo localization (GMCL), which integrates three particle filters into AMCL to reduce pose tracking errors and improve success rates in global localization and kidnapped robot scenarios [27]. However, existing MCL research primarily focuses on improving particle sampling efficiency and localization success rates. The particle state update in MCL-based robot localization algorithms relies on the robot’s movement displacement, meaning the robot must move to update its pose. When the movement displacement is small, the pose update efficiency is low. Furthermore, during the cold start phase, prolonged continuous movement is required to obtain a relatively accurate initial pose, leading to slow localization speed during the cold start phase.

In this paper, a new AMCL framework is proposed by introducing a virtual motion model. By using Normal Distributions Transform (NDT)-based point cloud matching, we estimate the deviation between the current pose and the actual pose, calculate the virtual displacement and virtual control quantities, and update the particle states based on the virtual control quantities. This allows for quick localization, even when the robot has not moved or has only moved a small distance, improving the localization speed during the cold start phase. Additionally, the EKF algorithm [28] is used to integrate IMU and encoder measurements of the robot’s motion information as the robot odometry motion model. This addresses the issue of odometry model deviations caused by encoder errors and wheel slippage, effectively improving localization stability and accuracy.

## 2. AMCL Algorithm

Monte Carlo localization (MCL) is applicable to both local and global localization problems. It updates the pose by incorporating appropriate probabilistic motion and perception models into the particle filter algorithm. A particle filter is a robust, non-parametric method used to address problems with multimodal belief distributions. This technique originates from Bayesian filtering and builds the posterior distribution through a resampling process. Particles with higher importance weights are more likely to be retained, as higher weight values increase the probability of a particle being selected. The belief, or the estimate of the robot’s current pose, is a probability density function. MCL uses a set of *M* particles $\chi_{t}=[{\vec{x}}_{t}^{\left[1\right]},{\vec{x}}_{t}^{\left[2\right]},\cdots {\vec{x}}_{t}^{\left[M\right]}]$ to represent the belief bel(${\vec{x}}_{t}$). Each particle is defined as a hypothetical pose ${\vec{x}}_{t}={\left[x_{t}^{r},y_{t}^{r},\theta_{t}^{r}\right]}^{T}$ of the robot, representing the robot’s position and yaw angle information in the reference coordinate system at time *t*. The weight of a particle represents the confidence level of the robot pose that the particle indicates. The initial confidence bel(${\vec{x}}_{0}$) is obtained by randomly generating *M* particles from a prior distribution $p({\vec{x}}_{0})$, while assigning the same importance factor $M^{-1}$ to each particle. As shown in Equations (1)–(4), the next state of all particles is predictively updated through the motion model. Then, through the measurement model, weights are calculated for the updated particles. Finally, particles with low weights are discarded, and new particles are randomly generated around the existing particles for repeated iteration. During the particle discarding process, all particles near the correct pose may accidentally be discarded. When the number of particles is small and they are distributed over a large range, the probability of discarding the correct particles increases. This situation can lead to failure in global localization or the kidnapped robot problem, and the system may not be able to recover from global localization. To address this issue, random particles can be added to the particle set for mitigation.

(1) $$\bar{bel}({\vec{x}}_{t})=\int p({\vec{x}}_{t}|u_{t},{\vec{x}}_{t-1})bel({\vec{x}}_{t-1})d{\vec{x}}_{t-1}$$

In the equation, $u_{t}$ represents the control input of the robot at time *t* and $\bar{bel}({\vec{x}}_{t})$ represents the prior belief at time *t*, which is a prediction of the robot’s pose at time *t* after it has moved under the control $u_{t}$ at time *t*, based on the posterior belief that $bel({\vec{x}}_{t-1})$ at time *t* − 1. $bel({\vec{x}}_{t-1})$ represents the posterior belief that at time *t* − 1. $p({\vec{x}}_{t}|u_{t},{\vec{x}}_{t-1})$ is the motion model.

(2) $$bel({\vec{x}}_{t-1})=\eta p(z_{t}|{\vec{x}}_{t})\bar{bel}({\vec{x}}_{t-1})$$

In the equation, η is the normalization constant, whose value is chosen in a way that the $bel({\vec{x}}_{t-1})$ sums up to one. $p(z_{t}|{\vec{x}}_{t})$ is a correction model used to update robot pose. The model will generate a measurement probability $p(z_{t}|{\vec{x}}_{t}^{\left[n\right]})$ for each particle ${\vec{x}}_{t}^{\left[n\right]}$. $z_{t}$ represents exteroceptive measurements.

MCL adds particles by monitoring the probability $p(z_{t}|z_{t-1},u_{t},m)$ of sensor measurements and comparing it with the average measurement probability. The average value can be noted as

(3) $$\frac{1}{M}\sum_{n=1}^{M}\omega_{t}^{\left[n\right]}\approx p(z_{t}|z_{t-1},u_{t},m)$$

In the equation, m is the map; $\omega_{t}^{\left[n\right]}$ as the weights of particles represents random approximation of this probability.

$\omega_{avg,t}$ is the average weight of all particles at time *t*, which can be expressed as

(4) $$\omega_{avg,t}=\omega_{avg,t-1}+\frac{1}{M}\sum_{n=1}^{M}\omega_{t}^{\left[n\right]}$$

As an improved algorithm of Monte Carlo localization, AMCL introduces random particles mathematically by assuming the robot is “kidnapped” with a small probability. The injection of these random particles creates a degree of random states in the motion model, and their presence can enhance the robustness of the system even when the robot is not actually kidnapped [25].

The AMCL algorithm determines if the robot is “kidnapped” based on the long-term estimated weight $\omega_{slow,t}$ and the short-term estimated weight $\omega_{fast,t}$ at time *t*. If $\omega_{slow,t}$ is greater than $\omega_{fast,t}$, it indicates that the robot is “kidnapped”. By adding random particles during resampling, the robot can recover from being “kidnapped”, which is expressed as follows.

(5) $$\left\{\begin{array}{l} \omega_{slow,t}=\omega_{slow,t-1}+\alpha_{slow}(\omega_{avg,t}-\omega_{slow,t-1})\\ \omega_{fast,t}=\omega_{fast,t-1}+\alpha_{fast}(\omega_{avg,t}-\omega_{fast,t-1}) \end{array}\right.$$

In the equation, $\omega_{avg,t}$ represents the average weight of all particles. The parameters $\alpha_{slow}$ and $\alpha_{fast}$ represent the decay rates of the exponential filters for estimating long-term and short-term averages, respectively, and $0\leq \alpha_{slow}\leq \alpha_{fast}$ is defined.

Additionally, AMCL uses Kullback–Leibler distance (KLD) [24] for adaptive adjustment of the number of particles during the resampling phase, which is expressed as follows:

(6) $$M_{\chi }=\frac{k-1}{2\varepsilon }{\left[1-\frac{2}{9(k-1)}+\sqrt{\frac{2}{9(k-1)}}z_{1-\delta }\right]}^{3}$$

In the equation, ε represents the error between the true distribution and the estimated distribution, $z_{1-\delta }$ denotes the 1−δ quantile on the standard normal distribution, and *k* represents the non-empty bins of the histogram. The upper limit of particles, $M_{\chi }$, has a linear relationship with the non-empty bins *k*.

**Figure 1:**
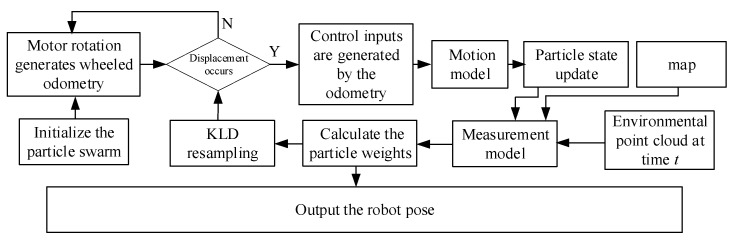
AMCL algorithm framework.

In the initial phase of global localization, all bins are non-empty, and the value of k increases with each new resampling, further leading to an increase in the upper limit of particles. After some time, once global localization is completed, the particles converge near the true pose, and the *k* value decreases; then, $M_{\chi }$ is reduced. This dynamic adjustment of the number of particles improves the computational efficiency of the algorithm. The AMCL algorithm framework is shown in Figure 1. At the start of the algorithm, the particle population is initialized, and the motion model is used to predictively update the population particles, representing the change in the robot’s pose after movement due to control input. Then, the measurement model is used to calculate the weights of the new population particles; higher weight values indicate that the particles are closer to the robot’s true pose, while particles with low weights will be removed from the population. Finally, new particles are introduced into the population through resampling, and the motion model is used to predictively update the new population particles, continuing the cycle.

However, the AMCL algorithm has the following issues:

1. During the robot’s localization process, the robot must be displaced through motion control for the AMCL algorithm to use the motion model for pose predictive updating.
2. Wheel slippage and drift can cause deviations in the motion information collected by the motor encoders, resulting in odometry measurement errors. Such errors can affect the predictive accuracy of the robot’s motion in the AMCL algorithm, leading to a decrease in localization accuracy.

## 3. Improved AMCL Algorithm

**Figure 2:**
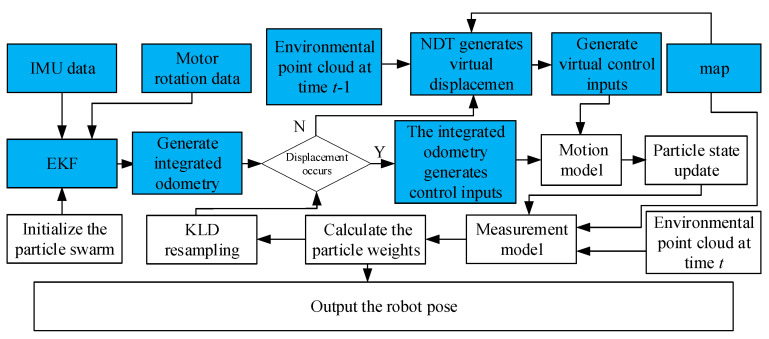
Framework of the improved AMCL algorithm.

To address the issue with the AMCL algorithm where particle states can only be updated through robot displacement via motion control, a Normal Distribution Transform (NDT) [29] algorithm is introduced to optimize the AMCL algorithm. The EKF algorithm [30] is used to integrate the motion information of the robot measured by the IMU and encoders as the robot’s odometry motion model, to address the issue of deviations in the odometry model caused by encoder errors and wheel slippage. The framework of the improved algorithm is shown in Figure 2. First, particles are initialized to distribute across the map space. The EKF is used to integrate IMU information and motor rotation data to generate fused odometry information. When the robot moves, the particle states are updated using the real displacement information. When the robot’s displacement is minimal, the NDT algorithm is used to generate virtual displacement, and the particle states are predictively updated based on this virtual displacement. The measurement model then calculates the particle weights, selecting particles that are close to the true pose. Finally, new particles are injected through KLD, and the above filtering process is repeated.

### 3.1. Virtual Motion Model Based on NDT

As a probabilistic point cloud matching method, the NDT point cloud matching algorithm maps point cloud data to a smoothed surface, represented as a set of local probability density functions (PDF). By optimizing the objective function, it iteratively obtains the relative pose difference between two frames of point clouds. In this paper, the environmental map is converted into a reference point cloud. The NDT algorithm is used to obtain the relative pose difference between the reference point cloud and the laser point cloud. This relative pose difference generates virtual control ${\hat{u}}_{t}$ and virtual displacement, further completing the particle state update step. The NDT generation ${\hat{u}}_{t}$ process is as follows.

Step 1: Convert the map into a point cloud and divide it into multiple grid cells, calculating the PDF for each cell.

(7) $$p(\vec{s})=\frac{1}{{\left(2\pi \right)}^{D/2}\sqrt{\left|\Sigma \right|}}\exp\left(-\frac{{\left(\vec{s}-\vec{\mu }\right)}^{T}\Sigma^{-1}(\vec{s}-\vec{\mu })}{2}\right)$$

In the equation, $\vec{\mu }$ and Σ represent the mean vector and covariance matrix of the points on the grid cell within the cell, as shown in Equations (8) and (9).

(8) $$\vec{\mu }=\frac{1}{m}\sum_{k=1}^{m}{\vec{s}}_{k}$$

(9) $$\Sigma =\frac{1}{m-1}\sum_{k=1}^{m}({\vec{s}}_{k}-\vec{\mu }){\left({\vec{s}}_{k}-\vec{\mu }\right)}^{T}$$

In the equation, ${\vec{s}}_{k=1,\ldots ,m}$ represents the position of the points within the grid cell.

Step 2: Set pose $\vec{pos}={\left[{\hat{x}}_{t},{\hat{y}}_{t},{\hat{\theta }}_{t}\right]}^{T}$ to map the scan point cloud Zt−1={Z→1,t−1…,Z→n}t−1 at time *t* − 1 to the map point cloud. The normal distribution of each grid cell after transformation is calculated using the following equation:

(10) p(T(pos→,Z→k)t−1)=−exp−(T(pos→,Z→k)t−1−μ→k)TΣk−1(T(pos→,Z→k)t−1−μ→k)2

In the equation, the transformation *T* represents the relationship of transformation between the point cloud scanned by the LiDAR at time *t* − 1 and the point cloud as converted to the map. $\Sigma_{k}^{-1}$ represents the inverse of the covariance matrix for the *k*-th grid cell in Equation (9). ${\vec{Z}}_{t-1}^{k}$ represents the vector representation of the point cloud within the *k*-th grid cell scanned by the LiDAR, centered on the robot.

Step 3: Use Equation (11) to calculate the probability density for each grid cell, which serves as the objective function for determining the optimal pose $\vec{pos}$.

(11) s(pos→)=−∑k=1nexp−(T(pos→,Z→k)t−1−μ→k)TΣk−1(T(pos→,Z→k)t−1−μ→k)2

Step 4: Obtain the objective function through Step 3 and use the Newton iteration algorithm to calculate the optimal pose $\vec{pos}$.

Step 5: Calculate the virtual control input ${\hat{u}}_{t}$ from the current robot pose ${\vec{x}}_{t-1}={\left[x_{t-1},y_{t-1},\theta_{t-1}\right]}^{T}$ to the optimal pose $\vec{pos}$.

(12) $$\left\{\begin{array}{l} \hat{\Delta }x={\hat{x}}_{t}-x_{t-1}\\ \hat{\Delta }y={\hat{y}}_{t}-y_{t-1}\\ \hat{\Delta }\theta ={\hat{\theta }}_{t}-\theta_{t-1} \end{array}\right.$$

(13) $${\hat{u}}_{t}={\left[\frac{\hat{\Delta }x}{\Delta t},\frac{\hat{\Delta }y}{\Delta t},\frac{\hat{\Delta }\theta }{\Delta t}\right]}^{T}$$

Generate ${\hat{u}}_{t}$ as the virtual control for when the robot has not moved and input it into the motion model to optimize and adjust the particles of the AMCL algorithm. Then, use the improved motion model to predictively update the optimized particles.

### 3.2. Odometry Motion Model Based on EKF Integration

During the robot’s movement, wheel speed information is typically collected through motor encoders to generate the odometry model. However, in practical applications, the odometry model may exhibit deviations due to encoder errors and wheel slippage. This inaccuracy can affect the results of the predictive update of the particles, causing them to deviate from the expected theoretical outcome, thereby reducing localization accuracy. Therefore, this paper uses the EKF algorithm to integrate the robot motion information measured by the IMU and the encoder as the robot odometry motion model. The specific steps are as follows.

Step 1: The robot pose prediction at time *t* is completed through Equation (14).

(14) $${\hat{\vec{x}}}_{t}=f({\vec{x}}_{t-1},u_{odo,t})=\left[\begin{array}{l} x_{t-1}+\Delta x_{odo,t}\cos\theta_{t-1}-\Delta y_{odo,t}\sin\theta_{t-1}\\ y_{t-1}+\Delta y_{odo,t}\sin\theta_{t-1}+\Delta y_{odo,t}\cos\theta_{t-1}\\ \theta_{t-1}+\Delta \theta_{odo,t} \end{array}\right]$$

In this equation, $x_{t-1},y_{t-1},\theta_{t-1}$ represents the position coordinates and yaw angle of the robot at time *t* − 1, while $u_{odo,t}={\left[\Delta x_{odo,t},\Delta y_{odo,t},\Delta \theta_{odo,t}\right]}^{T}$ represents the incremental displacement of the x,y axes and the change in yaw angle measured by wheeled odometer.

Step 2: The term $F_{t}$ denotes the Jacobian matrix of the function f at the t−1 instance. The derivative of function f with respect to ${\vec{x}}_{t-1}$ at $\left({\vec{x}}_{t-1},u_{odo,t}\right)$ is given by

(15) $$F_{t}=\frac{\partial f}{\partial \vec{x}}=\left[\begin{array}{ccc} 1 & 0 & -\Delta x_{odo,t}\sin\theta_{t-1}-\Delta y_{odo,t}\cos\theta_{t-1}\\ 0 & 1 & \Delta x_{odo,t}\cos\theta_{t-1}-\Delta y_{odo,t}\sin\theta_{t-1}\\ 0 & 0 & 1 \end{array}\right]$$

Step 3: Wheeled odometer noise error can be expressed as

(16) $$Q_{t}=\left[\begin{array}{ccc} \sigma^{2}\Delta x_{odo} & 0 & 0\\ 0 & \sigma^{2}\Delta y_{odo} & 0\\ 0 & 0 & \sigma^{2}\Delta \theta_{odo} \end{array}\right]$$

In this equation $\sigma \Delta x_{odo},\sigma \Delta y_{odo},\sigma \Delta \theta_{odo}$, respectively, represent the standard deviation of the *x* and *y*-axis displacement noise of the wheeled odometer and the standard deviation of the yaw angle noise.

Step 4: A prediction error covariance matrix ${\hat{P}}_{t}$, used to represent the uncertainty error of predicted pose values, can be described as

(17) $${\hat{P}}_{t}=F_{t}P_{t-1}F_{t}^{T}+Q_{t}$$

Step 5: The observation model is shown in the following formula, using IMU data as observational input.

(18) $$h({\vec{x}}_{t},{\vec{x}}_{t-1})=\left[\begin{array}{c} ({\vec{x}}_{t}-{\vec{x}}_{t-1})\cos\theta_{t-1}+({\vec{y}}_{t}-{\vec{y}}_{t-1})\sin\theta_{t-1}\\ -({\vec{x}}_{t}-{\vec{x}}_{t-1})\sin\theta_{t-1}+({\vec{y}}_{t}-{\vec{y}}_{t-1})\cos\theta_{t-1}\\ \theta_{t}-\theta_{t-1} \end{array}\right]$$

Step 6: The observation Jacobian matrix can be given by the following formula.

(19) $$H_{t}=\frac{\partial h}{\partial \vec{x}}=\left[\begin{array}{ccc} \cos\theta_{t-1} & \sin\theta_{t-1} & ({\vec{x}}_{t}-{\vec{x}}_{t-1})\cos\theta_{t-1}+({\vec{y}}_{t}-{\vec{y}}_{t-1})\sin\theta_{t-1}\\ -\sin\theta_{t-1} & \cos\theta_{t-1} & -({\vec{x}}_{t}-{\vec{x}}_{t-1})\sin\theta_{t-1}+({\vec{y}}_{t}-{\vec{y}}_{t-1})\cos\theta_{t-1}\\ 0 & 0 & 1 \end{array}\right]$$

Step 7: The Kalman gain is calculated through Equation (20).

(20) $$K_{t}={\hat{P}}_{t}H_{t}^{T}{\left(H{\hat{P}}_{t}H_{t}^{T}+R_{t}\right)}^{-1}$$

In the equation, $R_{t}$ represents the covariance of the IMU observation noise, given by the following formula:

(21) $$R_{t}=\left[\begin{array}{ccc} \sigma_{\Delta x_{imu}}^{2} & 0 & 0\\ 0 & \sigma_{\Delta y_{imu}}^{2} & 0\\ 0 & 0 & \sigma_{\Delta \theta_{imu}}^{2} \end{array}\right]$$

In the equation, $\sigma \Delta x_{imu},\sigma \Delta y_{imu},\sigma \Delta \theta_{imu}$, respectively, represent the standard deviation of the *x* and *y*-axis displacement noise of the IMU and the standard deviation of the yaw angle noise.

Step 8: The robot pose update at time *t* is completed through Equation (22).

(22) $${\vec{x}}_{t}={\hat{\vec{x}}}_{t}+K_{t}({\vec{z}}_{t}-h({\hat{\vec{x}}}_{t},{\vec{x}}_{t-1}))$$

In the equation, ${\vec{z}}_{t}={\left[\Delta x_{imu},\Delta y_{imu},\Delta \theta_{imu}\right]}^{T}$ represents the displacement increment of the robot’s coordinate system, calculated by the IMU and the change in the yaw angle measured by the IMU.

Step 9: The estimated error covariance matrix at time *t* is calculated through Equation (23).

(23) $$P_{t}=(I-K_{t}H_{t}){\hat{P}}_{t}$$

Step 10: Calculate the actual control input $u_{t}$ of the robot pose from time *t* − 1 to time *t*.

(24) $$\left\{\begin{array}{l} \Delta x=x_{t}-x_{t-1}\\ \Delta y=y_{t}-y_{t-1}\\ \Delta \theta =\theta_{t}-\theta_{t-1} \end{array}\right.$$

(25) $$u_{t}={\left[\frac{\Delta x}{\Delta t},\frac{\Delta y}{\Delta t},\frac{\Delta \theta }{\Delta t}\right]}^{T}$$

In the traditional AMCL algorithm, a particle filter cycle can only be performed when the scale of the robot’s movement reaches a certain threshold. If the scale of movement is small, or the robot undergoes small-scale pose changes, the algorithm does not update the robot’s pose. The improved localization algorithm incorporates an NDT process and optimizes the motion model to enhance the speed of particle convergence and positioning accuracy while improving system stability. This allows for pose correction and updates of the robot using scan information, even when the robot is not moving, thereby increasing localization accuracy and reducing localization time. The pseudocode for the NDT-based improved AMCL algorithm is as in Algorithm 1.
**Algorithm 1:** Improved AMCL Algorithm Based on NDT**Input:**The particle set from the previous time step $X_{t-1}$;
Current lidar scan point cloud $Z_{t}$;
Environmental map m;
Previous time-step lidar scan point cloud $Z_{t-1}$;
Total number of particles *M*;**Output:**Particle set at the current time step $X_{t}$;1:Static $\omega_{slow},\omega_{fast}$;2:${\bar{X}}_{t}=X_{t}=\emptyset$;3:**if** The robot has not undergone any displacement **then**4:      Calculate ${\hat{u}}_{t}$ according to Equation (13);5:      **for** *i* = 1 to *M* **do**6:            Update $x_{t}^{\left[i\right]}$ using ${\hat{u}}_{t}$ according to Equation (1);7:            Calculate the weight $\omega_{t}^{\left[i\right]}$ of each particle based on the map and lidar information;8:      **end for**9:**else**10:      **for** *i* = 1 to *M* **do**
11:            Update $x_{t}^{\left[i\right]}$ using $u_{t}$ according to Equation (1);12:            Calculate the weight $\omega_{t}^{\left[i\right]}$ of each particle based on the map and lidar information;13:      **end for**14:${\bar{X}}_{t}={\bar{X}}_{t}+<x_{t}^{\left[i\right]},\omega_{t}^{\left[i\right]}>$;15:Calculate $\omega_{slow},\omega_{fast}$;16:**for** *i* = 1 to *M* **do**17:**      with** probability $\max(0,0,1.0-\omega_{fast}/\omega_{slow})$**do**18:            add random pose to $X_{t}$;19:**      else**20:            draw $x_{t}^{\left[i\right]}$ from ${\bar{X}}_{t}$ with probability $\propto \omega_{t}^{\left[i\right]}$;21:**            **$X_{t}=X_{t}+<x_{t}^{\left[i\right]},\omega_{t}^{\left[i\right]}>$;22:**      end with**23:**end for**24:**return** $X_{t}$;

## 4. Results

The traditional AMCL algorithm for localization consists of two stages. In the first stage, the robot’s odometry trajectory information is used to obtain the pose transformation information of the robot between times *t* and *t* − 1. By calculating the trajectory between these two moments, the control inputs needed to transition the robot’s pose from *t* − 1 to *t* are determined. These control inputs are then fed into the motion model to predict the robot’s pose. In the second stage, point cloud data from the LIDAR scan at time *t* is used as posterior data to update the measurement of the first stage and pose prediction, completing the robot’s pose estimation after multiple iterative cycles. However, in the first stage, if the robot does not move, there is no trajectory information for computation, causing the localization process to remain in the first stage and increasing the localization duration. The improved AMCL algorithm proposed in this paper addresses localization when the robot’s initial pose is unknown. In the first stage, when the robot is stationary, point cloud data from the LIDAR scan at time *t* − 1 is used as prior data. The NDT algorithm generates virtual movement data for the robot, creating virtual control inputs for the time interval between *t* and *t* − 1, which are fed into the motion model to predict the robot’s pose, advancing the localization to the second stage. When the robot moves, cumulative errors in the wheel odometry can cause data drift, making the obtained pose transformation information from *t* to *t* − 1 inaccurate. This paper uses an EKF to fuse wheel odometry and IMU data to correct odometry errors. The corrected odometry is used to calculate the trajectory from time *t* − 1 to *t*, generating the control inputs needed for the robot’s pose transition from *t* − 1 to *t*, which are then used in the motion model for pose prediction, allowing the localization process to reach the second stage.

The primary improvements of the proposed AMCL algorithm focus on localization time and accuracy when the robot’s initial pose is unknown. To validate this, three sets of experiments were designed in both simulated and real environments, comparing the localization performance under the same conditions to the traditional AMCL, GMCL, and Cartographer algorithms.

### 4.1. Simulation Validation

**Figure 3:**
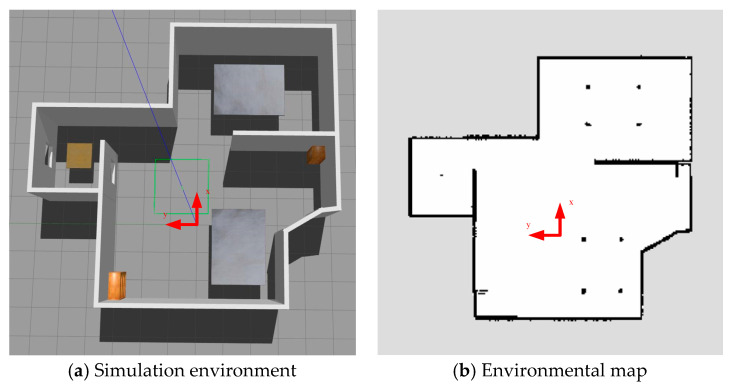
Simulation environment and map.

This paper compares the localization time and accuracy of the traditional AMCL algorithm, the improved AMCL algorithm, the GMCL algorithm, and the cartographer algorithm in ROS Noetic on Ubuntu 20.04. A simulation experiment is designed in the Gazebo 11.x software, adding models to simulate real-world physical and collision properties. The simulation environment is shown in Figure 3a, which includes items such as tables and bookshelves to simulate the real world. The world coordinate system of the simulation environment is indicated by a red arrow, with the arrow’s starting point representing the origin of the world coordinate system. The measurement range of the lidar is set to 6 m, with a resolution of 0.01 m and a scan frequency of 5.5 Hz. The update frequency of the IMU is 100 Hz. An environmental map is constructed using the Gmapping algorithm, as shown in Figure 3b. The map coordinate system is also indicated by a red arrow, with the arrow’s starting point as the origin of the map coordinate system. In the map, the white areas represent obstacle-free regions, black indicates obstacles, and gray represents unknown areas.

(1) Cold start localization time consumption test

**Figure 4:**
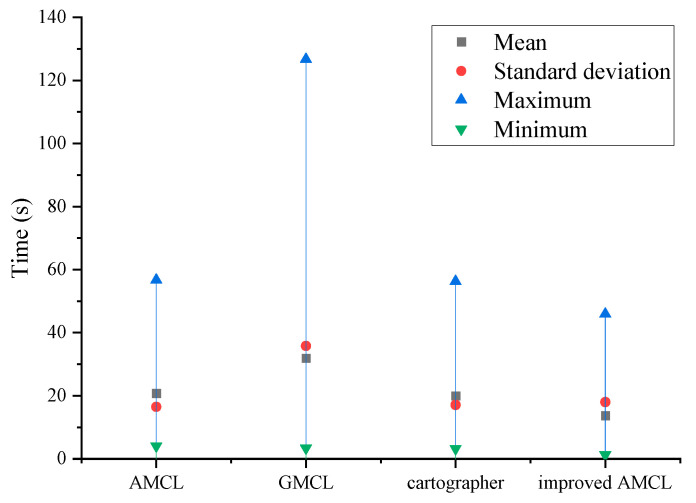
Comparison of robot localization time.

Start localization was set with the robot in different poses in the simulated world. The robot’s maximum linear velocity is 0.2 m/s, and the maximum angular velocity is 0.2 rad/s. The robot begins random motion from the initial position. Localization is considered successful if the robot’s positioning error is within 0.3 m and the localization yaw angle is within 15 degrees. The robot starts localization from different poses in the environment. Timing begins when the robot starts localization and ends when the robot completes localization. A total of 15 experiments are conducted, and the robot’s localization time is shown in Figure 4. The AMCL algorithm has an average localization time of 20.77 s, the GMCL algorithm has an average localization time of 31.88 s, the cartographer algorithm has an average localization time of 19.95 s, and the improved AMCL algorithm has an average localization time of 13.68 s.

(2) Pose accuracy test

**Figure 5:**
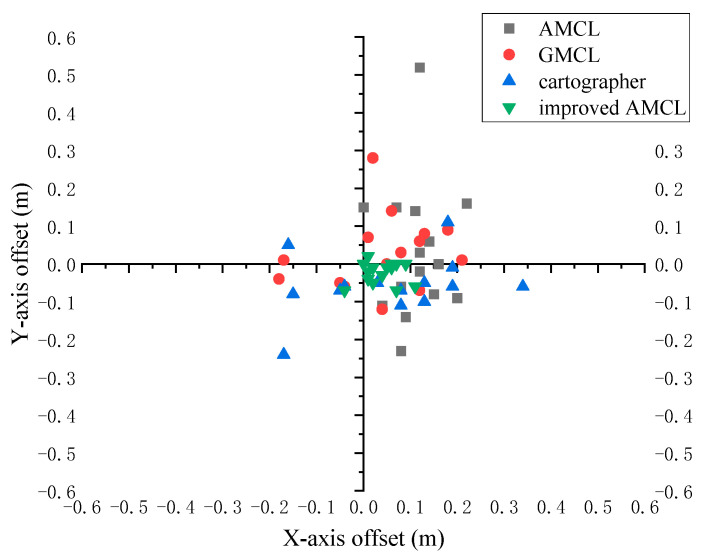
The offset of the localized position relative to the true position.

To further verify the localization effectiveness of the improved algorithm for the robot in dynamic scenarios, the robot could move randomly in the environment. The robot’s localization information and the actual information in the simulated environment were read at regular intervals. Data were read 15 times, and the displacement of the robot’s localized position relative to the actual position is shown in Figure 5. The coordinate point (0,0) indicates that the robot’s localized position coincides with the actual position, with an error of 0. The further a point deviates from the coordinate point (0,0) in the figure, the larger the localization error. The gray square points represent the positional offsets produced by the traditional AMCL algorithm, the red circular points indicate the offsets produced by the GMCL algorithm, the blue upward-pointing triangles represent the offsets produced by the cartographer algorithm, and the green downward-pointing triangles represent the offsets produced by the improved AMCL algorithm.

**Figure 6:**
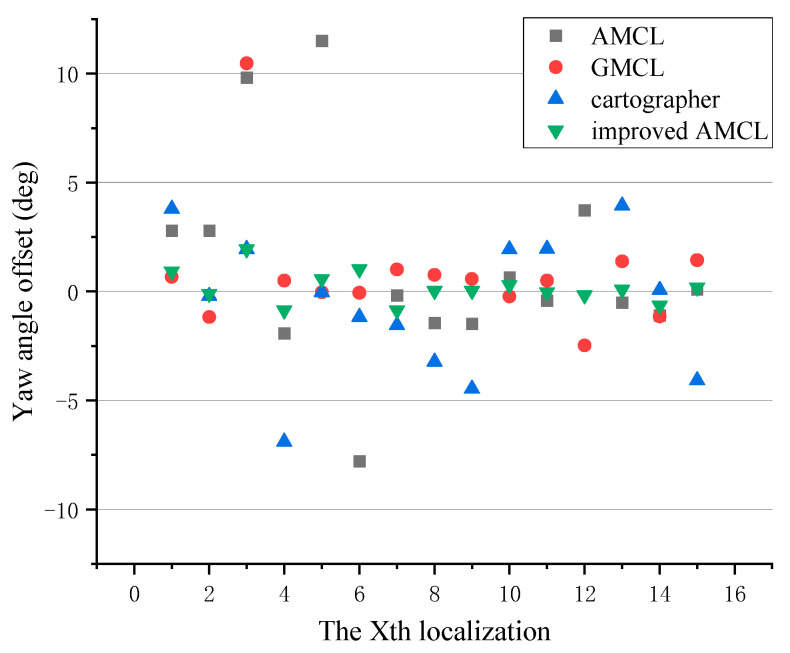
The offset of the localized yaw angle relative to the true yaw angle.

The yaw angle errors generated during the robot’s movement localization are shown in Figure 6. In the figure, points closer to a deviation of 0 indicate smaller localization errors. Taking the counterclockwise direction as the positive direction of the yaw angle, points above 0 indicate a positive deviation in the yaw angle of localization, while points below 0 indicate a negative deviation. The gray square points represent the yaw angle deviation produced by the traditional AMCL algorithm, the red circular points represent the deviation produced by the GMCL algorithm, the blue upward-pointing triangles represent the deviation produced by the cartographer algorithm, and the green downward-pointing triangles represent the deviation produced by the improved AMCL algorithm.

**Figure 7:**
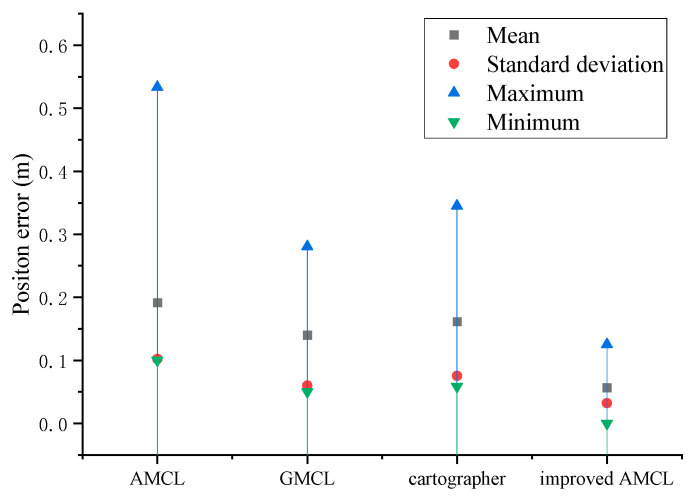
Position error statistics results.

**Figure 8:**
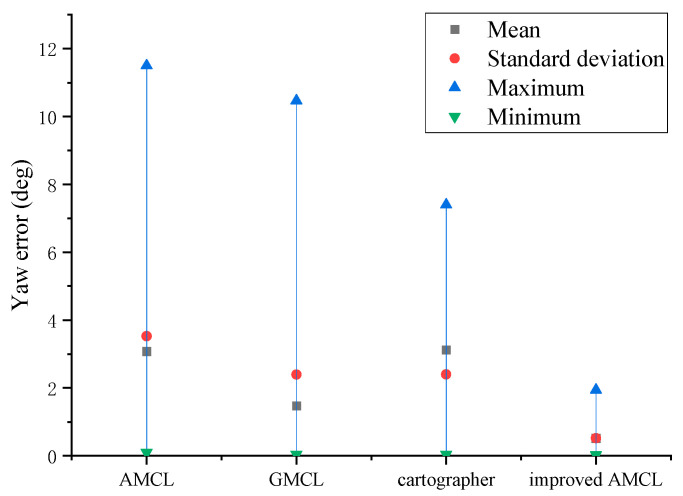
Yaw angle error statistics results.

The data statistics of Figure 5 and Figure 6 yield the results shown in Figure 7 and Figure 8, with the position error and yaw angle error given by Equation (26).

(26) $$\begin{array}{l} \Delta =\sqrt{\Delta x^{2}+\Delta y^{2}}\\ \delta =|\theta -\theta_{*}| \end{array}$$

In Equation (26), Δx,Δy represents the displacement of the robot’s localized position relative to the actual position using different algorithms and $\theta ,\theta_{*}$ represents the yaw angle of the robot localized by different algorithms and the actual yaw angle of the robot, respectively.

Through the data statistics of Figure 7 and Figure 8, it was observed that both the position error and the yaw angle error of the improved AMCL algorithm were reduced. Specifically, the average position error decreased from 0.12 m to 0.08 m, and the average yaw angle error decreased from 3.08 degrees to 0.51 degrees.

(3) Preset trajectory localization test

**Figure 9:**
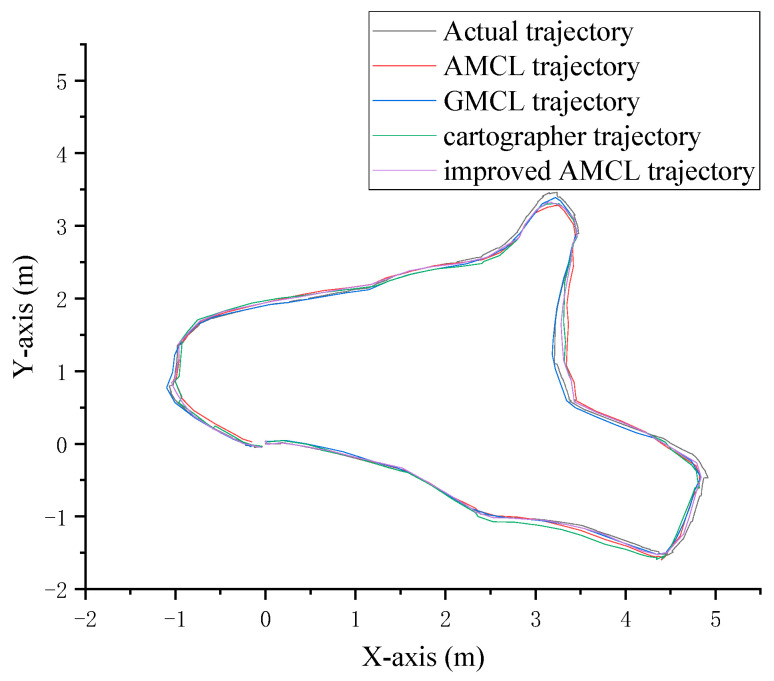
Robot motion trajectory and localization trajectory.

The robot was guided to follow a preset trajectory in the environment, and different algorithms were used to generate corresponding trajectories based on the robot’s localization data. The final trajectory curves are shown in Figure 9. The black curve represents the actual trajectory of the robot’s movement, with the real trajectory data provided by Gazebo. The red curve represents the motion trajectory generated by the traditional AMCL algorithm based on localization data, the blue curve represents the trajectory generated by the GMCL algorithm, the green curve represents the trajectory generated by the cartographer algorithm, and the purple curve represents the trajectory generated by the improved AMCL algorithm.

**Figure 10:**
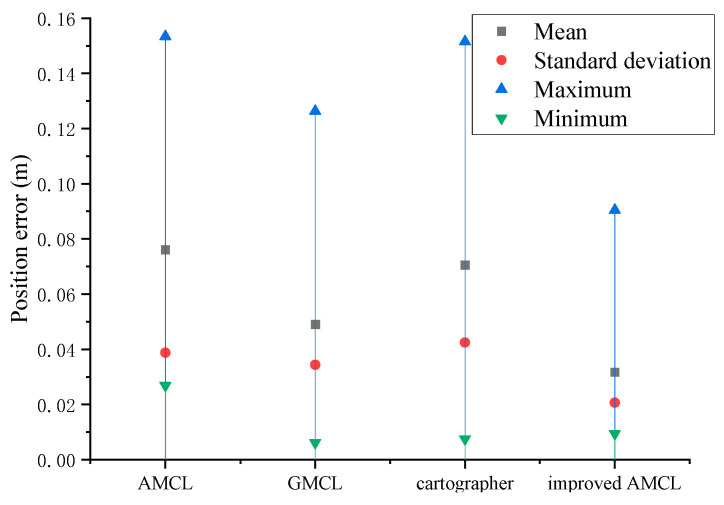
Trajectory error statistics results.

By performing data statistics on the localization trajectories of different algorithms shown in Figure 9 and calculating the position error using Equation (26), the statistical results are shown in Figure 10. When the robot follows a specific trajectory, the average position error for the improved AMCL algorithm is 0.024 m, the average deviation for the cartographer algorithm is 0.070 m, the average deviation for the GMCL algorithm is 0.050 m, and the average position error for the traditional AMCL algorithm is 0.064 m.

The three experiments described above demonstrate that the improved AMCL algorithm achieves reductions in both localization time and accuracy compared to the traditional AMCL algorithm, the GMCL algorithm, and the cartographer algorithm. Specifically, the minimum localization time has been reduced to 1.29 s, which is 2.81 s less than the original AMCL algorithm, 2.127 s less than the GMCL algorithm, and 1.95 s less than the cartographer algorithm. The average time has been reduced by 7 s, 18 s, and 6 s compared to the original AMCL, GMCL, and cartographer algorithms, respectively, and it has a smaller standard deviation, meaning localization times are more concentrated around the mean. The improved AMCL algorithm reduces the average positional error to below 0.057 m, which is an improvement of 0.133 m, 0.083 m, and 0.103 m compared to the original AMCL’s 0.19 m, GMCL’s 0.14 m, and cartographer’s 0.16 m, respectively. The average yaw angle error is reduced to 0.519 degrees, which is 2.561 degrees less than the 3.08 degrees of the original AMCL algorithm, and the data are more concentrated around the mean. This indicates that in a simulation environment, the improved AMCL algorithm shows enhancements in localization time and pose accuracy.

### 4.2. Real-World Environment Experiment

**Figure 11:**
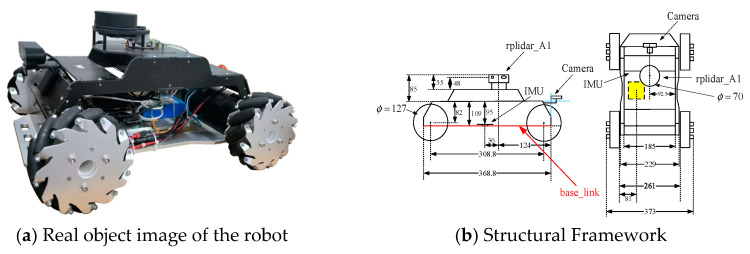
Photographs and structural diagrams of the robot.

To further test the localization performance of the improved algorithm, a robot, as shown in Figure 11, was constructed. The structural diagram below illustrates the distribution of the robot’s key sensors and the position of the robot’s body coordinate system (base_link) within the robot. The main sensor parameters of the robot are as follows. The LiDAR uses the SLAM-based RPLIDAR A1, which provides high-precision environmental sensing capabilities. The IMU uses the ICM20948, which can provide real-time motion status and directional information. Regarding the motors, the robot uses standard DC motors equipped with TB6112 driver chips and Hall encoders, ensuring stable power output and precise position control.

For ease of expression during the localization process, the pose information of the base_link coordinate system will be used as the robot’s pose information. The positions of the robot’s LiDAR, IMU, and camera will be provided based on their relative position information with respect to the base_link. The point cloud information $z_{t}^{k}$ scanned by the LiDAR is transformed into the base_link coordinate system for processing, as shown in Equation (27).

(27) $$\left[\begin{array}{c} x_{z_{t}^{k}}\\ y_{z_{t}^{k}} \end{array}\right]=\left[\begin{array}{c} x_{base\_link}\\ y_{base\_link} \end{array}\right]+\left[\begin{array}{cc} \cos\theta & -\sin\theta \\ \sin\theta & \cos\theta \end{array}\right]\left[\begin{array}{c} x_{k,rplidar}\\ y_{k,rplidar} \end{array}\right]+z_{t}^{k}\left[\begin{array}{c} \cos(\theta +\theta_{k,rplidar})\\ \sin(\theta +\theta_{k,rplidar}) \end{array}\right]$$

**Figure 12:**
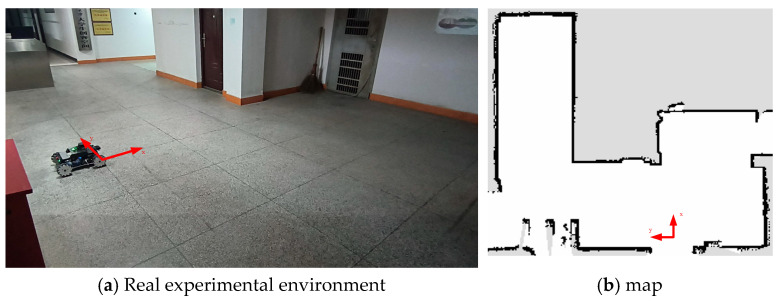
Real experimental environment and map.

The experimental environment is chosen as shown in Figure 12a, located in an irregular room with an area of approximately 35 square meters. The red coordinate system in the figure is defined as the map coordinate system and is used as the reference coordinate system for the robot’s localization. The robot’s pose is represented as pose information in the map coordinate system. An environmental map, as shown in Figure 12b, is constructed based on the real environment.

(1) Cold start localization time consumption test

**Figure 13:**
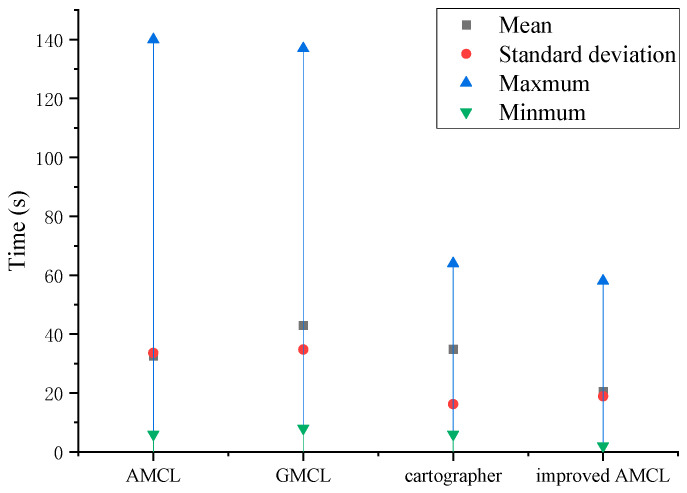
Comparison of robot localization time.

In the experiment, the robot’s initial pose was set to be unknown, with a maximum linear velocity of 0.2 m/s and a maximum angular velocity of 0.2 rad/s. The robot started localization from different poses in the environment. Localization was considered complete when the error between the robot’s localized position and the actual position was less than 0.3 m, and the error between the localized yaw angle and the actual yaw angle was less than 15 degrees. The robot began localization from different poses in the environment, and timing started when the robot began localization and ended when localization was complete. Fifteen experiments were conducted, and the robot’s localization time is shown in Figure 13. The traditional AMCL algorithm has an average localization time of 32.56 s, the GMCL algorithm has an average localization time of 43 s, the cartographer localization algorithm has an average localization time of 34.86 s, and the improved AMCL algorithm has an average localization time of 20.56 s.

(2) Pose accuracy test

**Figure 14:**
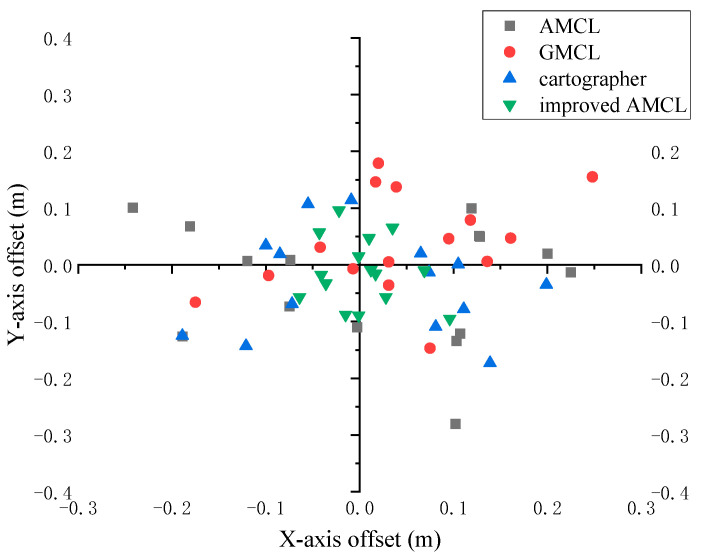
The offset of the localized position relative to the true position.

To verify the localization performance of the robot during movement, a mobile localization experiment was designed. The robot randomly moved in the environment, and after moving for a certain period, the robot’s current actual pose was measured and compared with the pose information obtained through the localization algorithm. Through fifteen data readings, the displacement of the robot’s localized position relative to the actual position is shown in Figure 14. In the figure, the center of the coordinate axis represents the actual position, and the farther away from the center, the greater the localization error. Gray squares represent the displacement when the robot uses the traditional AMCL for localization, red dots represent the displacement when using GMCL, the blue upward-pointing triangles represent the displacement when using cartographer, and the green downward-pointing triangles represent the displacement when using improved AMCL.

**Figure 15:**
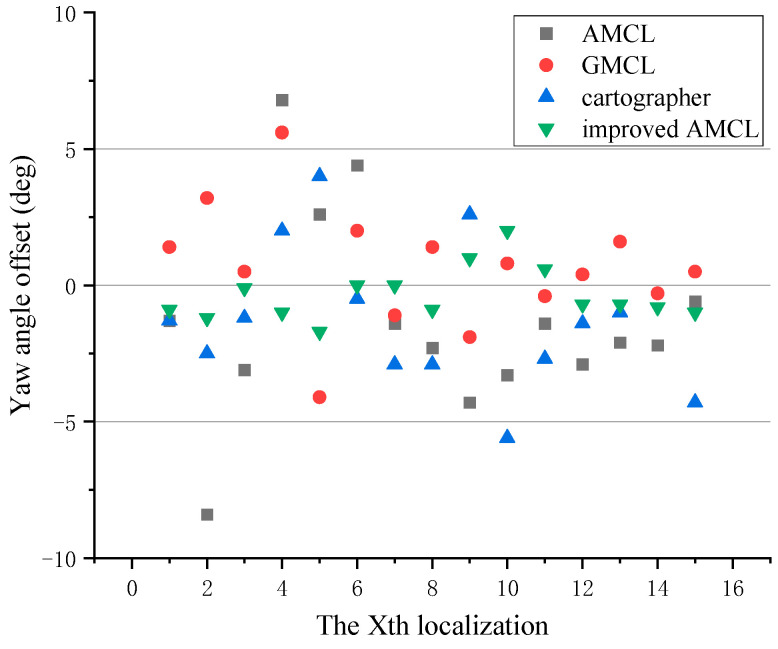
The offset of the localized yaw angle relative to the true yaw angle.

The yaw angle errors of different algorithms are shown in Figure 15, with the counterclockwise direction being positive and the clockwise direction being negative. A difference greater than zero indicates that the localized yaw angle is positively deviated from the actual yaw angle, while a difference less than zero indicates a negative deviation. In the figure, gray squares represent the difference between the yaw angle localized by the traditional AMCL algorithm and the robot’s actual yaw angle, red dots represent the difference for the GMCL algorithm, blue upward-pointing triangles represent the difference for the cartographer algorithm, and green downward-pointing triangles represent the difference for the improved AMCL algorithm. The closer the difference in the figure is to zero, the more accurate the yaw angle localization of the algorithm.

**Figure 16:**
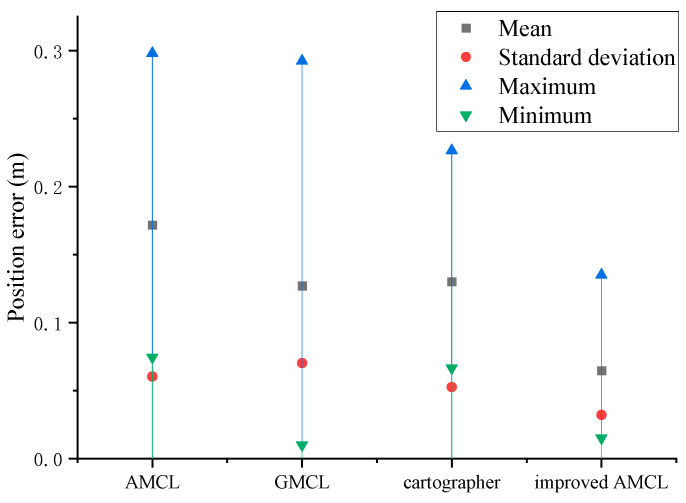
Position error statistics chart.

**Figure 17:**
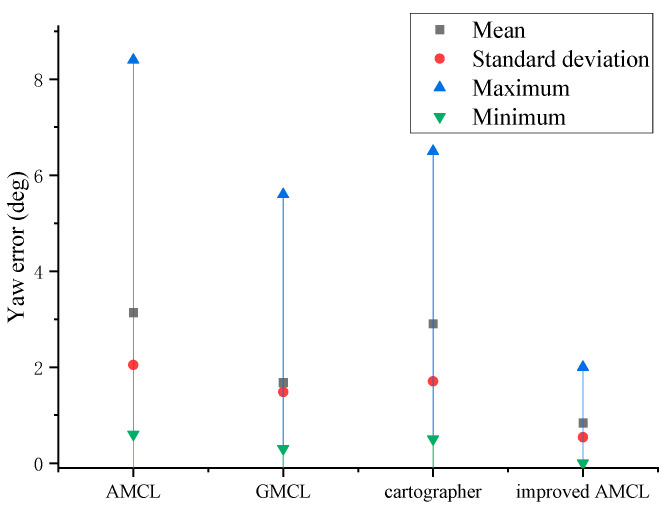
Yaw angle error statistics chart.

Based on the data statistics from Figure 14 and Figure 15, the results shown in Figure 16 and Figure 17 are obtained, with the position error and yaw angle error given by Equation (17). The improved AMCL algorithm has an average positioning error of 0.06 m and an average yaw angle error of 0.84 degrees. The cartographer algorithm has an average positioning error of 0.13 m and an average yaw angle error of 2.9 degrees. The GMCL algorithm’s average positioning error is 0.12 m, with an average yaw angle error of 1.68 degrees. The original AMCL algorithm has an average positioning error of 0.17 m and an average yaw angle error of 3.14 degrees.

(3) Preset trajectory localization test

**Figure 18:**
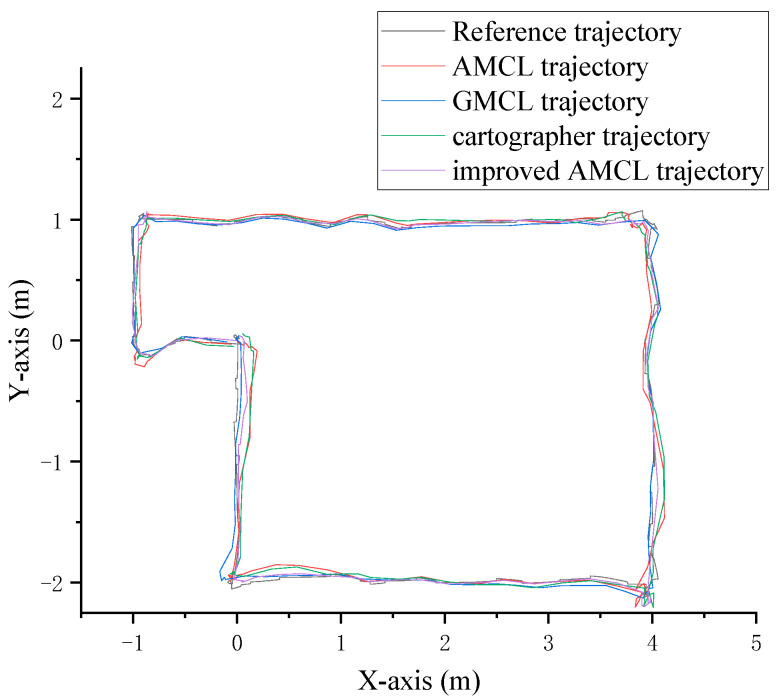
Robot motion trajectory and localization trajectory.

The robot walks along a preset trajectory in the real environment, and different algorithms generate corresponding trajectories based on the robot’s localization data. The final trajectory curves are shown in Figure 18. In the figure, the black curve represents the reference preset trajectory during the robot’s movement, the red curve represents the motion trajectory generated by the traditional AMCL algorithm based on localization data, the blue curve represents the motion trajectory generated by the GMCL algorithm, the green curve represents the motion trajectory generated by the cartographer algorithm, and the purple curve represents the motion trajectory generated by the improved AMCL algorithm.

**Figure 19:**
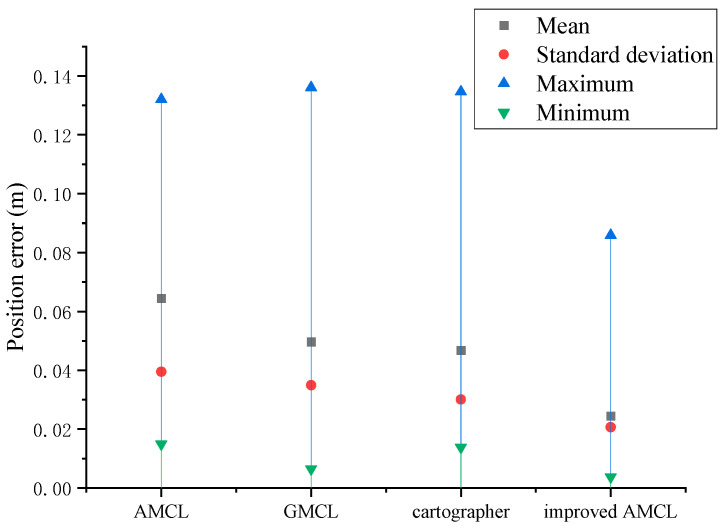
Trajectory error statistics chart.

The data statistics of the different localization trajectory algorithms in Figure 18 were analyzed, and the localization position error was calculated using Equation (26). The statistical results are shown in Figure 19. When the robot walks along a specific trajectory, the average localization displacement is 0.031 m for the improved AMCL algorithm, 0.076 m for the traditional AMCL algorithm, 0.049 m for the GMCL algorithm, 0.046 m and for the cartographer algorithm.

The three experiments described above demonstrate that the improved AMCL algorithm achieves reductions in both localization time and accuracy compared to the traditional AMCL algorithm, the GMCL algorithm, and the cartographer algorithm. Specifically, the minimum localization time decreased to 2 s, which is 4 s less compared to the traditional AMCL algorithm, 6 s less compared to the GMCL algorithm, and 4 s less compared to cartographer algorithm. The average time decreased by 12 s, 23 s, and 14 s compared to the traditional AMCL, the GMCL algorithm, and cartographer algorithms, respectively, with a smaller standard deviation, indicating that the localization times are more concentrated around the mean value. The improved AMCL algorithm reduces the average positioning error to below 0.06 m, which is 0.11 m, 0.06 m, and 0.07 m less than the original AMCL algorithm’s 0.17 m, the GMCL algorithm’s 0.12 m, and cartographer’s 0.13 m, respectively. The average yaw angle error is reduced to 0.84 degrees, decreasing by 2.3 degrees compared to the original AMCL algorithm’s 3.14 degrees, by 0.84 degrees compared to the GMCL algorithm’s 1.68 degrees, and by 2.06 degrees compared to cartographer’s 2.90 degrees. Additionally, the data are more concentrated around the mean. This indicates that in real environments, the improved AMCL algorithm shows advancements in localization time and pose accuracy.

## 5. Conclusions

This paper investigates the issues of localization accuracy and efficiency of mobile robots under the ROS system. An improved AMCL algorithm is proposed to address the problem of excessive localization time of the traditional AMCL algorithm when the robot’s initial pose is unknown. This algorithm, while inheriting the high robustness of the AMCL algorithm, incorporates NDT and EKF as enhancements. NDT uses the environment map as reference information by converting the environment map into a reference point cloud, and the LiDAR scan data as the source data, transforming the LiDAR scan data into an input point cloud and obtaining the transformation matrix between point clouds using the Newton iteration method. EKF enhances the system’s ability to assess changes in the robot’s pose by integrating motor encoder data and IMU data, resulting in a more accurate motion model. Experimental results show that in the Gazebo simulation environment, the improved algorithm reduces the average localization time by 7 s and increases the average localization accuracy by 0.133 m when the robot’s initial pose is unknown. In a real-world scenario, the improved algorithm reduces the average localization time by 4 s and increases the average localization accuracy by 0.11 m under unknown initial pose conditions.

## Data Availability

Data is unavailable due to privacy restriction.
